# Hexaaqua­cadmium(II) 2,2′-(azino­dimethyl­idyne)dibenzene­sulfonate dihydrate

**DOI:** 10.1107/S1600536808039032

**Published:** 2008-11-26

**Authors:** Lian-Cai Du

**Affiliations:** a College of Bioengineering, Weifang University, Weifang 261061, People’s Republic of China

## Abstract

In the title compound, [Cd(H_2_O)_6_](C_14_H_10_O_6_N_2_S_2_)·2H_2_O, the complete cation and anion are each generated by crystallographic inversion symmetry. In the crystal structure, the components form a three-dimensional network by way of O—H⋯O and O—H⋯N hydrogen bonds.

## Related literature

For background to the properties and potential applications of organic–inorganic hybrid materials, see: Hagrman *et al.* (1998 ▶); Ranford *et al.* (1998 ▶).
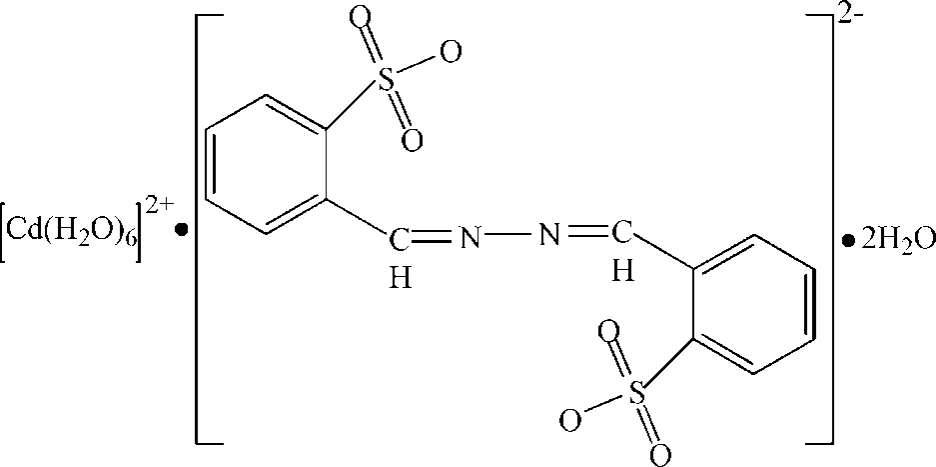


## Experimental

### 

#### Crystal data

[Cd(H_2_O)_6_](C_14_H_10_O_6_N_2_S_2_)·2H_2_O
*M* *_r_* = 622.89
Triclinic, 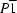

*a* = 7.8329 (11) Å
*b* = 7.9824 (12) Å
*c* = 10.1010 (15) Å
α = 92.723 (1)°
β = 102.076 (2)°
γ = 105.924 (2)°
*V* = 590.19 (15) Å^3^
*Z* = 1
Mo *K*α radiation
μ = 1.17 mm^−1^
*T* = 298 (2) K
0.45 × 0.40 × 0.28 mm

#### Data collection

Bruker SMART CCD diffractometer
Absorption correction: multi-scan (*SADABS*; Bruker, 2000 ▶) *T*_min_ = 0.621, *T*_max_ = 0.735
3081 measured reflections
2041 independent reflections
1929 reflections with *I* > 2σ(*I*)
*R*_int_ = 0.018

#### Refinement

*R*[*F* ^2^ > 2σ(*F* ^2^)] = 0.023
*wR*(*F* ^2^) = 0.061
*S* = 1.06
2041 reflections
152 parameters
H-atom parameters constrained
Δρ_max_ = 0.33 e Å^−3^
Δρ_min_ = −0.42 e Å^−3^

### 

Data collection: *SMART* (Bruker, 2000 ▶); cell refinement: *SAINT* (Bruker, 2000 ▶); data reduction: *SAINT*; program(s) used to solve structure: *SHELXS97* (Sheldrick, 2008 ▶); program(s) used to refine structure: *SHELXL97* (Sheldrick, 2008 ▶); molecular graphics: *SHELXTL* (Sheldrick, 2008 ▶); software used to prepare material for publication: *SHELXTL*.

## Supplementary Material

Crystal structure: contains datablocks global, I. DOI: 10.1107/S1600536808039032/hb2858sup1.cif

Structure factors: contains datablocks I. DOI: 10.1107/S1600536808039032/hb2858Isup2.hkl

Additional supplementary materials:  crystallographic information; 3D view; checkCIF report

## Figures and Tables

**Table 1:** Selected bond lengths (Å)

Cd1—O5	2.2555 (18)
Cd1—O4	2.2589 (17)
Cd1—O6	2.2947 (18)

**Table 2:** Hydrogen-bond geometry (Å, °)

*D*—H⋯*A*	*D*—H	H⋯*A*	*D*⋯*A*	*D*—H⋯*A*
O4—H4*A*⋯O2^i^	0.85	1.94	2.783 (3)	171
O4—H4*B*⋯O1^ii^	0.85	2.03	2.872 (2)	174
O5—H5*A*⋯O1^iii^	0.85	1.99	2.831 (3)	173
O5—H5*B*⋯O7^iv^	0.85	2.00	2.843 (3)	173
O6—H6*A*⋯O7	0.85	2.08	2.881 (3)	157
O6—H6*B*⋯N1^v^	0.85	2.15	2.993 (3)	169
O7—H7*A*⋯O3^vi^	0.85	1.85	2.692 (3)	172
O7—H7*B*⋯O2^iii^	0.85	2.21	3.002 (3)	156

Symmetry codes: (i) 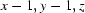; (ii) 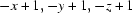; (iii) 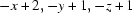; (iv) 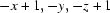; (v) 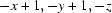; (vi) 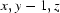.

**Fig. 1.**
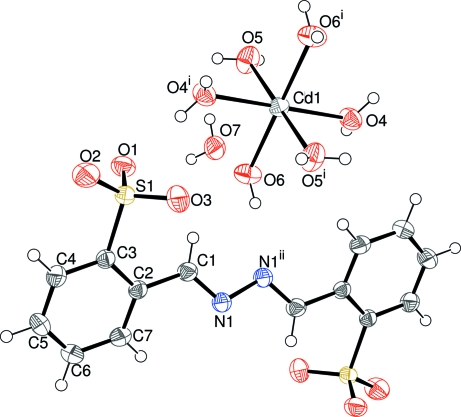
The molecular structure of (I) showing 50% displacement ellipsoids for the non-hydrogen atoms. Symmetry codes: (i) 1–x, 1–y, 1–z; (ii) 1–x, 1–y, –z.

## supplementary crystallographic information

### Comment

The design and synthesis of organic/inorganic hybrid materials have attracted intense attention in recent years owing to their potential practical applications, such as antitumor, antidiabetic, antitubercular activities, magnetism and catalysis [Ranford, *et al.*, 1998; Hagrman, *et al.*, 1998].   As part of our studies in this area, we now report the synthesis and crystal structure of the title compound, (I).

The Cd(II) centre is six-coordinate with six O donors of H_2_O, and adopts distorted octahedral coordination (Table 1, Fig. 1).  In the crystal, the molecules form a three-dimensional network by way of O—H···O and O—H···N hydrogen bonds (Table 2).

### Experimental

A solution of 1.0 mmol 2-formyl-benzenesulfonic acid-hydrazine and 1.0 mmol NaOH in 5 ml 95% ethanol was added to a solution of 0.5 mmol Cd(CH_3_COO)_2_.4H_2_O in 5 ml ethanol at room temperature. The mixture was refluxed for 4 h with stirring, then the resulting precipitate was filtered, washed, and dried *in vacuo* over P_4_O_10_ for 48 h. Colourless blocks of (I) were obtained by slowly evaporating from methanol at room temperature.

### Refinement

H atom treatment??

### Crystal data

**Table:**

[Cd(H_2_O)_6_](C_14_H_10_O_6_N_2_S_2_)·2H_2_O	*Z* = 1
*M**_r_* = 622.89	*F*_000_ = 316
Triclinic, *P*1	*D*_x_ = 1.753 Mg m^−^^3^
Hall symbol: -P 1	Mo *K*α radiation λ = 0.71073 Å
*a* = 7.8329 (11) Å	Cell parameters from 2719 reflections
*b* = 7.9824 (12) Å	θ = 2.7–28.3º
*c* = 10.1010 (15) Å	µ = 1.17 mm^−^^1^
α = 92.723 (1)º	*T* = 298 (2) K
β = 102.076 (2)º	Block, colourless
γ = 105.924 (2)º	0.45 × 0.40 × 0.28 mm
*V* = 590.19 (15) Å^3^	

### Data collection

**Table:**

Bruker SMART CCD diffractometer	2041 independent reflections
Radiation source: fine-focus sealed tube	1929 reflections with *I* > 2σ(*I*)
Monochromator: graphite	*R*_int_ = 0.018
*T* = 298(2) K	θ_max_ = 25.0º
ω scans	θ_min_ = 2.1º
Absorption correction: multi-scan(SADABS; Bruker, 2000)	*h* = −9→9
*T*_min_ = 0.621, *T*_max_ = 0.735	*k* = −5→9
3081 measured reflections	*l* = −11→12

### Refinement

**Table:**

Refinement on *F*^2^	Hydrogen site location: inferred from neighbouring sites
Least-squares matrix: full	H-atom parameters constrained
*R*[*F*^2^ > 2σ(*F*^2^)] = 0.023	*w* = 1/[σ^2^(*F*_o_^2^) + (0.0313*P*)^2^ + 0.2007*P*] where *P* = (*F*_o_^2^ + 2*F*_c_^2^)/3
*wR*(*F*^2^) = 0.061	(Δ/σ)_max_ = 0.001
*S* = 1.06	Δρ_max_ = 0.33 e Å^−^^3^
2041 reflections	Δρ_min_ = −0.42 e Å^−^^3^
152 parameters	Extinction correction: SHELXL97 (Sheldrick, 2008), Fc^*^=kFc[1+0.001xFc^2^λ^3^/sin(2θ)]^-1/4^
Primary atom site location: structure-invariant direct methods	Extinction coefficient: 0.067 (3)
Secondary atom site location: difference Fourier map	

### Special details

**Table:**

Geometry. All e.s.d.'s (except the e.s.d. in the dihedral angle between two l.s. planes) are estimated using the full covariance matrix. The cell e.s.d.'s are taken into account individually in the estimation of e.s.d.'s in distances, angles and torsion angles; correlations between e.s.d.'s in cell parameters are only used when they are defined by crystal symmetry. An approximate (isotropic) treatment of cell e.s.d.'s is used for estimating e.s.d.'s involving l.s. planes.
Refinement. Refinement of *F*^2^ against ALL reflections. The weighted *R*-factor *wR* and goodness of fit *S* are based on *F*^2^, conventional *R*-factors *R* are based on *F*, with *F* set to zero for negative *F*^2^. The threshold expression of *F*^2^ > σ(*F*^2^) is used only for calculating *R*-factors(gt) *etc*. and is not relevant to the choice of reflections for refinement. *R*-factors based on *F*^2^ are statistically about twice as large as those based on *F*, and *R*- factors based on ALL data will be even larger.

### Fractional atomic coordinates and isotropic or equivalent isotropic displacement parameters (Å^2^)

**Table:**

	*x*	*y*	*z*	*U*_iso_*/*U*_eq_	
Cd1	0.5000	0.5000	0.5000	0.02922 (13)	
N1	0.5732 (3)	0.5473 (3)	−0.0282 (2)	0.0336 (5)	
O1	1.0298 (2)	0.6986 (2)	0.35433 (17)	0.0384 (4)	
O2	1.1817 (3)	1.0036 (3)	0.35043 (19)	0.0465 (5)	
O3	0.8508 (3)	0.8917 (3)	0.2790 (2)	0.0477 (5)	
O4	0.2044 (2)	0.3385 (2)	0.45818 (18)	0.0406 (4)	
H4A	0.1847	0.2342	0.4229	0.049*	
H4B	0.1378	0.3362	0.5150	0.049*	
O5	0.6085 (3)	0.3128 (3)	0.6297 (2)	0.0471 (5)	
H5A	0.7201	0.3195	0.6358	0.057*	
H5B	0.5490	0.2060	0.6283	0.057*	
O6	0.5347 (3)	0.3561 (3)	0.30977 (19)	0.0448 (5)	
H6A	0.5293	0.2485	0.3123	0.054*	
H6B	0.4908	0.3730	0.2289	0.054*	
O7	0.6167 (3)	0.0337 (3)	0.3703 (2)	0.0468 (5)	
H7A	0.6908	−0.0043	0.3358	0.056*	
H7B	0.6594	0.0466	0.4560	0.056*	
S1	1.02370 (7)	0.85670 (8)	0.28844 (6)	0.02847 (16)	
C1	0.7223 (3)	0.5998 (3)	0.0615 (2)	0.0303 (5)	
H1	0.7234	0.5806	0.1517	0.036*	
C2	0.8948 (3)	0.6913 (3)	0.0237 (2)	0.0265 (5)	
C3	1.0408 (3)	0.8087 (3)	0.1183 (2)	0.0258 (5)	
C4	1.2027 (3)	0.8863 (3)	0.0822 (3)	0.0348 (6)	
H4	1.2984	0.9650	0.1451	0.042*	
C5	1.2224 (4)	0.8469 (4)	−0.0476 (3)	0.0423 (6)	
H5	1.3316	0.8989	−0.0717	0.051*	
C6	1.0809 (3)	0.7311 (4)	−0.1414 (3)	0.0386 (6)	
H6	1.0948	0.7044	−0.2284	0.046*	
C7	0.9177 (3)	0.6542 (3)	−0.1061 (2)	0.0337 (5)	
H7	0.8223	0.5768	−0.1701	0.040*	

### Atomic displacement parameters (Å^2^)

**Table:**

	*U*^11^	*U*^22^	*U*^33^	*U*^12^	*U*^13^	*U*^23^
Cd1	0.03192 (17)	0.02725 (17)	0.02920 (17)	0.01001 (10)	0.00609 (10)	0.00611 (10)
N1	0.0251 (10)	0.0408 (12)	0.0293 (11)	0.0001 (9)	0.0060 (8)	0.0066 (9)
O1	0.0449 (10)	0.0429 (10)	0.0318 (9)	0.0163 (8)	0.0123 (8)	0.0108 (8)
O2	0.0477 (11)	0.0433 (11)	0.0375 (10)	−0.0004 (9)	0.0074 (8)	−0.0093 (9)
O3	0.0429 (10)	0.0629 (13)	0.0486 (12)	0.0291 (10)	0.0168 (9)	0.0054 (10)
O4	0.0398 (10)	0.0394 (10)	0.0389 (10)	0.0022 (8)	0.0151 (8)	0.0000 (8)
O5	0.0416 (10)	0.0394 (11)	0.0614 (13)	0.0147 (9)	0.0069 (9)	0.0211 (9)
O6	0.0639 (12)	0.0415 (11)	0.0329 (10)	0.0200 (10)	0.0132 (9)	0.0038 (8)
O7	0.0447 (11)	0.0518 (12)	0.0501 (12)	0.0243 (9)	0.0123 (9)	−0.0007 (9)
S1	0.0288 (3)	0.0306 (3)	0.0257 (3)	0.0085 (2)	0.0063 (2)	0.0008 (2)
C1	0.0290 (12)	0.0316 (13)	0.0265 (12)	0.0036 (10)	0.0048 (10)	0.0033 (10)
C2	0.0251 (11)	0.0282 (12)	0.0260 (12)	0.0080 (9)	0.0044 (9)	0.0069 (9)
C3	0.0253 (11)	0.0268 (12)	0.0258 (12)	0.0086 (9)	0.0054 (9)	0.0055 (9)
C4	0.0259 (12)	0.0389 (14)	0.0329 (13)	0.0008 (10)	0.0040 (10)	0.0036 (11)
C5	0.0325 (13)	0.0565 (17)	0.0374 (15)	0.0052 (12)	0.0158 (11)	0.0127 (13)
C6	0.0401 (14)	0.0504 (16)	0.0287 (13)	0.0143 (12)	0.0131 (11)	0.0086 (12)
C7	0.0335 (13)	0.0382 (14)	0.0265 (12)	0.0087 (11)	0.0029 (10)	0.0021 (10)

### Geometric parameters (Å, °)

**Table:**

Cd1—O5^i^	2.2555 (18)	O6—H6B	0.8500
Cd1—O5	2.2555 (18)	O7—H7A	0.8499
Cd1—O4^i^	2.2589 (17)	O7—H7B	0.8500
Cd1—O4	2.2589 (17)	S1—C3	1.783 (2)
Cd1—O6^i^	2.2947 (18)	C1—C2	1.485 (3)
Cd1—O6	2.2947 (18)	C1—H1	0.9300
N1—C1	1.270 (3)	C2—C7	1.390 (3)
N1—N1^ii^	1.431 (4)	C2—C3	1.403 (3)
O1—S1	1.4621 (19)	C3—C4	1.382 (3)
O2—S1	1.4523 (19)	C4—C5	1.384 (4)
O3—S1	1.4414 (18)	C4—H4	0.9300
O4—H4A	0.8500	C5—C6	1.377 (4)
O4—H4B	0.8500	C5—H5	0.9300
O5—H5A	0.8500	C6—C7	1.385 (4)
O5—H5B	0.8500	C6—H6	0.9300
O6—H6A	0.8499	C7—H7	0.9300
			
O5^i^—Cd1—O5	180.0	O3—S1—O1	111.68 (12)
O5^i^—Cd1—O4^i^	95.45 (7)	O2—S1—O1	111.24 (12)
O5—Cd1—O4^i^	84.55 (7)	O3—S1—C3	106.73 (11)
O5^i^—Cd1—O4	84.55 (7)	O2—S1—C3	106.56 (11)
O5—Cd1—O4	95.45 (7)	O1—S1—C3	105.47 (10)
O4^i^—Cd1—O4	180.0	N1—C1—C2	120.7 (2)
O5^i^—Cd1—O6^i^	89.86 (7)	N1—C1—H1	119.7
O5—Cd1—O6^i^	90.14 (7)	C2—C1—H1	119.7
O4^i^—Cd1—O6^i^	90.19 (7)	C7—C2—C3	118.4 (2)
O4—Cd1—O6^i^	89.81 (7)	C7—C2—C1	119.8 (2)
O5^i^—Cd1—O6	90.14 (7)	C3—C2—C1	121.7 (2)
O5—Cd1—O6	89.86 (7)	C4—C3—C2	120.5 (2)
O4^i^—Cd1—O6	89.81 (7)	C4—C3—S1	118.63 (17)
O4—Cd1—O6	90.19 (7)	C2—C3—S1	120.88 (17)
O6^i^—Cd1—O6	180.0	C3—C4—C5	120.0 (2)
C1—N1—N1^ii^	111.5 (2)	C3—C4—H4	120.0
Cd1—O4—H4A	113.7	C5—C4—H4	120.0
Cd1—O4—H4B	123.5	C6—C5—C4	120.3 (2)
H4A—O4—H4B	108.3	C6—C5—H5	119.9
Cd1—O5—H5A	116.0	C4—C5—H5	119.9
Cd1—O5—H5B	123.2	C5—C6—C7	120.0 (2)
H5A—O5—H5B	108.7	C5—C6—H6	120.0
Cd1—O6—H6A	116.9	C7—C6—H6	120.0
Cd1—O6—H6B	123.6	C6—C7—C2	120.8 (2)
H6A—O6—H6B	109.5	C6—C7—H7	119.6
H7A—O7—H7B	105.6	C2—C7—H7	119.6
O3—S1—O2	114.51 (12)		
			
N1^ii^—N1—C1—C2	176.2 (2)	O3—S1—C3—C2	−47.1 (2)
N1—C1—C2—C7	−29.2 (4)	O2—S1—C3—C2	−169.90 (19)
N1—C1—C2—C3	154.3 (2)	O1—S1—C3—C2	71.8 (2)
C7—C2—C3—C4	0.5 (3)	C2—C3—C4—C5	−0.7 (4)
C1—C2—C3—C4	177.1 (2)	S1—C3—C4—C5	177.7 (2)
C7—C2—C3—S1	−177.85 (18)	C3—C4—C5—C6	0.3 (4)
C1—C2—C3—S1	−1.2 (3)	C4—C5—C6—C7	0.3 (4)
O3—S1—C3—C4	134.5 (2)	C5—C6—C7—C2	−0.5 (4)
O2—S1—C3—C4	11.7 (2)	C3—C2—C7—C6	0.1 (4)
O1—S1—C3—C4	−106.6 (2)	C1—C2—C7—C6	−176.6 (2)

Symmetry codes: (i) −*x*+1, −*y*+1, −*z*+1; (ii) −*x*+1, −*y*+1, −*z*.

### Hydrogen-bond geometry (Å, °)

**Table:**

*D*—H···*A*	*D*—H	H···*A*	*D*···*A*	*D*—H···*A*
O4—H4A···O2^iii^	0.85	1.94	2.783 (3)	171
O4—H4B···O1^i^	0.85	2.03	2.872 (2)	174
O5—H5A···O1^iv^	0.85	1.99	2.831 (3)	173
O5—H5B···O7^v^	0.85	2.00	2.843 (3)	173
O6—H6A···O7	0.85	2.08	2.881 (3)	157
O6—H6B···N1^ii^	0.85	2.15	2.993 (3)	169
O7—H7A···O3^vi^	0.85	1.85	2.692 (3)	172
O7—H7B···O2^iv^	0.85	2.21	3.002 (3)	156

Symmetry codes: (iii) *x*−1, *y*−1, *z*; (i) −*x*+1, −*y*+1, −*z*+1; (iv) −*x*+2, −*y*+1, −*z*+1; (v) −*x*+1, −*y*, −*z*+1; (ii) −*x*+1, −*y*+1, −*z*; (vi) *x*, *y*−1, *z*.
